# Transcriptom and miRNA data of PUFA-enriched stimulated murine macrophage and human endothelial cell lines

**DOI:** 10.1038/s41597-023-02288-8

**Published:** 2023-06-10

**Authors:** Claudia Roessler, Julia Schumann

**Affiliations:** University Clinic and Outpatient Clinic for Anaesthesiology and Operative Intensive Care, University Medicine Halle (Saale), 06112 Halle (Saale), Germany

**Keywords:** Bacterial infection, Toll-like receptors, miRNA in immune cells

## Abstract

Inflammation is associated with the adaptation of macrophages and endothelial cells, and the dysregulation of these differentiation processes has been directly linked to both acute and chronic disease states. As cells in constant contact with blood, macrophages and endothelial cells are also under the direct influence of immunomodulatory dietary components such as polyunsaturated fatty acids (PUFA). RNA sequencing analyses allow us to understand the global changes in gene expression occurring during cell differentiation, including both transcriptional (transcriptome) and post-transcriptional (miRNAs) levels. We generated a comprehensive RNA sequencing dataset of parallel transcriptome and miRNA profiles of PUFA-enriched and pro-inflammatory stimulated macrophages and endothelial cells aiming to uncover the underlying molecular mechanisms. PUFA concentrations and duration of supplementation were based on dietary ranges, allowing for metabolism and plasma membrane uptake of fatty acids. The dataset may serve as a resource to study transcriptional and post-transcriptional changes associated with macrophage polarisation and endothelial dysfunction in inflammatory settings and their modulation by omega-3 and omega-6 fatty acids.

## Background & Summary

Macrophages and endothelial cells are key cellular mediators of innate immune defence. Macrophages are directly activated by contact with bacterial surface structures, such as lipopolysaccharide (LPS; Gram-negative bacteria) or lipoteichoic acid (LTA; Gram-positive bacteria), initiating macrophage differentiation. There is a continuum of states of pro-inflammatory activation, often simplified as a differentiation to the pro-inflammatory M1 type^1^. Activation of endothelial cells is mediated by pro-inflammatory cytokines such as interleukin-1beta (IL-1β), tumour necrosis factor-alpha (TNF-α), and interferon-gamma (IFN-γ), which are released by M1-type macrophages^2^. The regulation of these processes is essential to combat pathogenic organisms and to maintain physical integrity. In fact, aberrant macrophage differentiation and endothelial dysfunction have been implicated in many acute and chronic diseases of inflammatory pathogenesis such as sepsis or artherosclerosis^3–5^.

Cellular differentiation and adaptive processes are accompanied by changes in protein expression. Inflammatory mediators initiate signal transduction cascades that ultimately influence the transcription of specific protein-coding genes. Besides bacterial surface structures and cytokines, polyunsaturated fatty acids (PUFA) influence gene expression in macrophages and endothelial cells^6^. Both cell types are particularly susceptible to dietary influences because they are in constant contact with the blood. Epidemiologic and interventional studies show protective effects of PUFA against adverse cardiovascular events, reduction of arterial stiffness and vascular inflammatory processes, and even improvements in sepsis patients^7^. Interestingly, PUFA may be modulators of gene expression through multiple pathways. Described mechanisms of action include (1) binding of PUFA to the immune cell receptors peroxisome proliferator-activated receptor gamma (PPARγ) and G protein-coupled receptor 120 (GPR120), (2) conversion of PUFA to eicosanoids and resolvins, which are potent immune mediators, and (3) incorporation of PUFA into the lipid raft domains of the plasma membrane, thereby affecting protein-protein interactions of membrane receptors^8^.

Besides transcriptional regulation, mRNA copy number is fine-tuned post-transcriptionally^9^. This is mediated by small non-coding RNAs, so-called miRNAs. miRNAs interact with mRNAs via partial complementarity, negatively affecting the stability and translational efficiency of targeted mRNAs^9^. Thus, a comprehensive understanding of protein expression requires a concurrent analysis of transcriptome and miRNAs. An efficient approach to global transcriptome and miRNA profiling is next-generation sequencing (NGS). It allows hypothesis-free, genome-wide analysis of mRNA and miRNA expression and their bioinformatics evaluation.

**Fig. 1:**
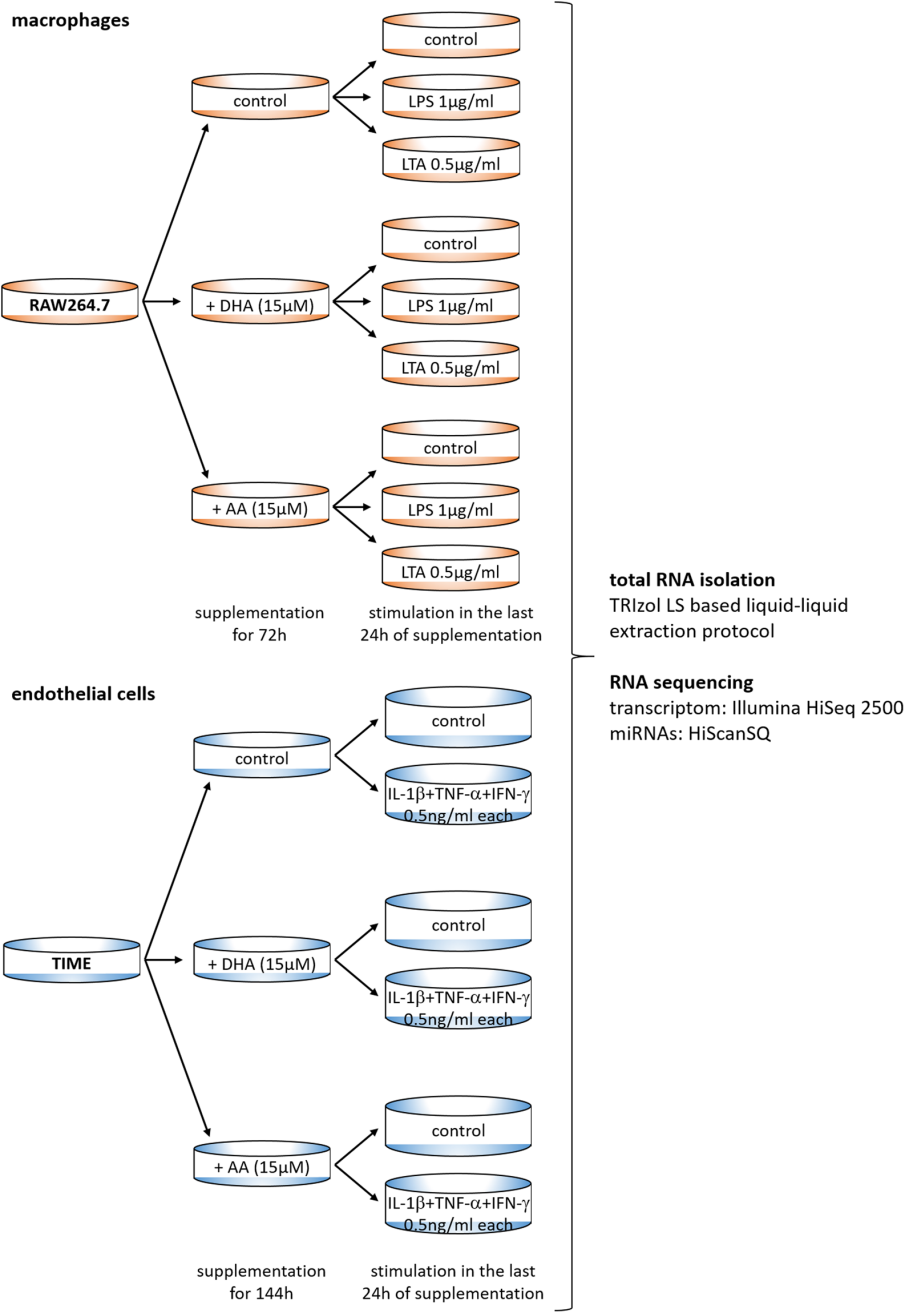
Study design and sampling standard. PUFA supplementation was performed by means of docosahexaenoic acid (DHA, C22:6n3) or arachidonic acid (AA, C20:4n6). Unsupplemented cells served as controls. Cell stimulation was performed during the last 24 h of PUFA supplementation. In the macrophage cell line, either lipopolysaccharide (LPS) or lipoteichoic acid (LTA) was used. For the endothelial cell line, a cytokine mixture consisting of IL-1β, TNF-α, and IFN-γ was added to stimulate the cells. Non-stimulated cells were included as controls. There were 3 biological replicates for each test group.

Genomics data is one of the fastest growing areas of big data. However, parallel transcriptome and miRNA profiling is still rare. This is especially true for analysing PUFA effects in the immunological setting. In this data descriptor, we aimed to provide both transcriptomic and miRNA data sets from inflammation-prone macrophages and endothelial cells supplemented with PUFA in a defined manner. As shown in the study design (Fig. 1), (1) naive versus LPS- or LTA-stimulated macrophages and (2) unstimulated versus cytokine-stimulated endothelial cells were analysed. The influence of the PUFA docosahexaenoic acid (DHA, an omega-3 fatty acid) and arachidonic acid (AA, an omega-6 fatty acid) was investigated by considering all possible combinations of fatty acid supplementation and stimulation. The duration of PUFA supplementation was chosen to allow incorporation of the fatty acids into the plasma membrane to reach a membrane steady state^10–12^. It is emphasized that supplementation was performed at a physiologically relevant concentration^13^. Preliminary work of the group shows that such PUFA enrichment of macrophage membrane interferes with the interaction of LPS-inducible Toll-like receptor 4 (TLR4) with its co-receptor CD14, which also favours macrophage differentiation to the anti-inflammatory M2 type in inflammatory settings^14–17^. PUFA administration also has anti-inflammatory effects in endothelial cells. Both stimulus-induced synthesis and release of inflammatory cytokines and the expression of adhesion proteins, which are important for macrophage trans-endothelial migration, are reduced in endothelial cells enriched in PUFA^12^. Therefore, the data set generated here may help other (nutritional) scientists and clinicians to gain new insights into how PUFA act. This dataset is intended to provide a useful and reliable tool to elucidate molecular mechanisms of PUFA modulation of macrophage/endothelial cell activation at both transcriptional and post-transcriptional levels. Two key representatives of omega-3 and omega-6 fatty acids, DHA and AA, were specifically selected to allow parallel and comparative studies of the effects of PUFA of these two subclasses.

## Methods

### Cell culture

Two commercially available cell lines were used: the murine macrophage cell line RAW264.7 (ATCC number: TIB-71) and the human telomerase-immortalized microvascular endothelial cell line TIME (ATCC number: CRL-4025). To ensure cell line integrity, both cell lines were purchased directly from LGC Standards, Wesel, Germany and the cell culture was regularly checked for mycoplasma contamination by PCR according to the recommendations of the Leibniz Institute DSMZ-German Collection of Microorganisms and Cell Cultures.

Cell culture medium used for RAW264.7 was RPMI 1640 (PANBiotech, Aidenbach, Germany) enriched with 5% v/v FCS (Invitrogen/Thermo Fischer Scientific, Dreieich, Germany). TIME were cultured in basal microvascular endothelial cell growth medium enriched with 5 ng/ml VEGF, 5 ng/ml EGF, 5 ng/ml FGF, 15 ng/ml IGF-1, 10 mM L-glutamine, 0.75 U/ml heparin sulphate, 1 μg/ml hydrocortisone hemisuccinate, 50 μg/ml ascorbic acid (all Provitro, Berlin, Germany), 5% v/v FCS, and 12.5 μg/ml blasticidin (Invitrogen/Thermo Fischer Scientific, Dreieich, Germany).

For cellular fatty acid enrichment RAW264.7 as well as TIME were incubated in cell culture medium supplemented with either docosahexaenoic acid (DHA, C22:6n3) or arachidonic acid (AA, C20:4n6) (both Biotrend, Köln, Germany) in concentrations of 15 μmol/l using ethanol as a vehicle (0.2% v/v final ethanol concentration). Cells were cultured in the enriched media in 75 cm^2^ cell culture flasks totaling either 72 h (RAW264.7) or 144 h (TIME) at 37 °C and 5% CO_2_ in a humidified atmosphere.

For stimulation RAW264.7 were treated with either 1 μg/ml LPS (from *Escherichia coli* serotype 0111:B4) or 0.5 µg/ml LTA (from *Staphylococcus aureus*) (Sigma-Aldrich, Taufkirchen, Germany) and TIME were treated with a cytokine mix consisting of IL-1β, TNF-α and IFN-γ, each in a concentration of 5 ng/ml (all PeproTech, Hamburg, Germany). Stimulation was performed in the last 24 hours of fatty acid supplementation.

Periods of supplementation and stimulation were proven to result in a membrane fatty acid steady state as well as reproducible effects on macrophage/endothelial cell functionality^10–12,15–17^.

### Total RNA isolation

Total RNA extraction was performed using a standard liquid–liquid extraction protocol based on TRIzol LS (Thermo Fisher Scientific, Dreieich, Germany) according to the manufacturer’s instructions. The concentration and quality of RNA gained were analyzed by means of the NanoDrop spectrophotometer (Thermo Fisher Scientific, Dreieich, Germany) as well as the Agilent Bioanalyzer (Agilent Technologies, Waldbronn, Germany).

### RNA sequencing

Total RNA samples (amount ≥ 0,8 µg) were submitted to commercial sequencing facilities for Illumina Next Generation Sequencing (NGS). mRNA sequencing was performed by the Novogene (UK) Company Limited utilizing an Illumina HiSeq 2500 (San Diego, CA, USA). Small RNA sequencing was performed both by the Novogene (UK) Company Limited and the Core Unit DNA, Leipzig University utilizing an Illumina HiScanSQ (San Diego, CA, USA). Sequencing libraries were prepared according to Illumina’s instructions accompanying the NEB Next Ultra RNA Library Prep Kit (Novogene) or the TruSeq Small RNA Prep kit v2 (Core Unit DNA, Leipzig). Three biological replicates were analyzed in each test group.

Processing of RNA-seq data was performed by the Core Facility Imaging, University Medicine Halle (Saale) for Novogene-analyzed samples and the Core Unit DNA, Leipzig University, respectively. The following steps were taken by the Core Facility Imaging, University Medicine Halle (Saale): Low quality read ends as well as remaining parts of sequencing adapters were clipped off using Cutadapt (v 1.14) with parameters -q 20 -O 7 -m 20. Trimmed reads were mapped against the mouse genome (mm10 UCSC) for cell line RAW264.7 or human genome (hg38 UCSC) for cell line TIME using (i) Bowtie2 (v 2.3.2) with parameters -N 1 for small RNA and (ii) HiSat2 (v 2.1.0) with parameters -p 6–dta–strandness RF -k 5 for poly-A-RNA, respectively. Secondary alignments were filtered out using samtools (v 1.5). Mapped reads were summarized using featureCounts (v 1.5.3). TMM normalisation was done using the R/Bioconductor package EdgeR. The following steps were carried out by the Core Unit DNA, Leipzig University: Basecalls and demultiplexing was performed using CASAVA version 1.4. Raw reads were adapter trimmed with cutadapt version 1.9.1. Only adapter trimmed reads between 15 and 27 bp lenght were considered processed miRNAs and selected for alignment. The small RNA seq reads were aligned to the mouse genome (mm10 UCSC) for cell line RAW264.7 or human genome (hg38 UCSC) for cell line TIME using Bowtie2 version 2.2.7 allowing 1 mismatch and alignment to multiple targets. Reads were annotated by intersecting genome coordinates of known miRNAs from miRBase version 21 using Bedtools version 2.25.0. Reads were counted using the R/Bioconductor programming environment by application of the ShortRead library and the table function together with the miRNA name in the annotation. Count normalisation was done using the R/Bioconductor packages DESeq 2 and EdgeR.

All generated RNA-seq data were deposited at the Gene Expression Omnibus (GEO) repository.

### RNA-seq data analyses

RNA-seq data were analyzed for differential gene expression, focusing on altered expressed miRNAs. Gene expression profiling was performed by the Core Facility Imaging, University Medicine Halle (Saale) for Novogene-analyzed samples and the Core Unit DNA, Leipzig University, respectively. Identified miRNA candidates were validated by Droplet Digital PCR (ddPCR) technology (BioRad, Munich, Germany). ddPCR allows determination of the absolute copy number of a miRNA per total amount of RNA in the sample. The need to refer to an (presumed) stably expressed gene, the so-called housekeeper, is omitted. This is particularly important in the inflammatory setting, as frequently used housekeepers, such as U6, have been shown to reveal changes in expression profile under inflammatory conditions making them non-suitable^18–20^.

The experimental studies were further augmented by *in silico* analyses. The target genes of differentially expressed miRNAs were identified using the online databases miRWalk2.0 (http://mirwalk.umm.uni-heidelberg.de)^21^ and DIANA-TarBase v8 (https://dianalab.e-ce.uth.gr/html/diana/web/index.php?r=tarbasev8/index)^22^. Gene ontology (GO) enrichment analysis was performed for the identified target genes of each miRNA using the online GeneOntology enrichment analysis and visualization tool (GOrilla; http://cbl-gorilla.cs.technion.ac.il)^23^ and the Cytoscape plugin ClueGO v2.5.7 (https://apps.cytoscape.org/apps/cluego)^24^, respectively. Also, Gene Set Enrichment Analyses (GSEA) of mRNA-seq data were performed by the Core Facility Imaging of the University Medicine Halle (Saale) to identify statistically significant, consistent differences in the transcriptome of stimulated versus unstimulated endothelial cells and macrophages, respectively.

A more detailed description of each of these analyses, as well as the results obtained, have been published in several articles^25–29^.

## Data Records

RNA-seq data files in FastQ format were deposited in the Gene Expression Omnibus (GEO) database under accession numbers GSE132361, GSE141957, GSE142088, GSE162994, and GSE162995^30–34^ These datasets contain 102 samples in total, grouped by 36 transcriptome subsets and 30 miRNA subsets from macrophages (cell line RAW264.7, ATCC number TIB-71) as well as 18 transcriptome subsets and 18 miRNA subsets from endothelial cells (cell line TIME, ATCC number CRL-4025). The sample information is summarised in Table 1.

**Table 1:** Sample details of RNA-seq profiling.

Source	Treatment	Molecule	Sample count	GEO Accession ID
RAW264.7	not supplemented, not stimulated	poly-A-RNA	6	GSE142088, GSE162994
RAW264.7	DHA supplemented, not stimulated	poly-A-RNA	6	GSE142088, GSE162994
RAW264.7	AA supplemented, not stimulated	poly-A-RNA	6	GSE142088, GSE162994
RAW264.	not supplemented, LPS stimulated	poly-A-RNA	3	GSE142088
RAW264.7	DHA supplemented, LPS stimulated	poly-A-RNA	3	GSE142088
RAW264.7	AA supplemented, LPS stimulated	poly-A-RNA	3	GSE142088
RAW264.7	not supplemented, LTA stimulated	poly-A-RNA	3	GSE162994
RAW264.7	DHA supplemented, LTA stimulated	poly-A-RNA	3	GSE162994
RAW264.7	AA supplemented, LTA stimulated	poly-A-RNA	3	GSE162994
RAW264.7	not supplemented, not stimulated	small RNA	6	GSE132361, GSE162995
RAW264.7	DHA supplemented, not stimulated	small RNA	3	GSE132361
RAW264.7	AA supplemented, not stimulated	small RNA	3	GSE132361
RAW264.7	not supplemented, LPS stimulated	small RNA	3	GSE132361
RAW264.7	DHA supplemented, LPS stimulated	small RNA	3	GSE132361
RAW264.7	AA supplemented, LPS stimulated	small RNA	3	GSE132361
RAW264.7	not supplemented, LTA stimulated	small RNA	3	GSE162995
RAW264.7	DHA supplemented, LTA stimulated	small RNA	3	GSE162995
RAW264.7	AA supplemented, LTA stimulated	small RNA	3	GSE162995
TIME	not supplemented, not stimulated	poly-A-RNA	3	GSE141957
TIME	DHA supplemented, not stimulated	poly-A-RNA	3	GSE141957
TIME	AA supplemented, not stimulated	poly-A-RNA	3	GSE141957
TIME	not supplemented, cytokine stimulated	poly-A-RNA	3	GSE141957
TIME	DHA supplemented, cytokine stimulated	poly-A-RNA	3	GSE141957
TIME	AA supplemented, cytokine stimulated	poly-A-RNA	3	GSE141957
TIME	not supplemented, not stimulated	small RNA	3	GSE132361
TIME	DHA supplemented, not stimulated	small RNA	3	GSE132361
TIME	AA supplemented, not stimulated	small RNA	3	GSE132361
TIME	not supplemented, cytokine stimulated	small RNA	3	GSE132361
TIME	DHA supplemented, cytokine stimulated	small RNA	3	GSE132361
TIME	AA supplemented, cytokine stimulated	small RNA	3	GSE132361

AA = arachidonic acid (C20:4n6), DHA = docosahexaenoic acid (C22:6n3), LPS = lipopolysaccharide, LTA = lipoteichoic acid.

## Technical Validation

### Cell line identity

Cell lines were purchased from the American Type Culture Collection (ATCC) through the distributor LGC Standards (Wesel, Germany). Only low passage (<10 passages) cells that were free of mycoplasma were used to obtain RNA samples for NGS analyses. Cell cultivation was performed in strict compliance with ATCC recommendations.

### RNA integrity

The quality of the isolated RNA was checked by NanoDrop spectrophotometer (Thermo Fisher Scientific, Dreieich, Germany) and Agilent 2100 Bioanalyzer (Agilent Technologies,Waldbronn, Germany) to meet the requirements for NGS analysis in terms of purity (absorbance quotient A260/280) and integrity (RNA integrity number (RIN)).

### RNA-seq raw data quality

**Fig. 2:**
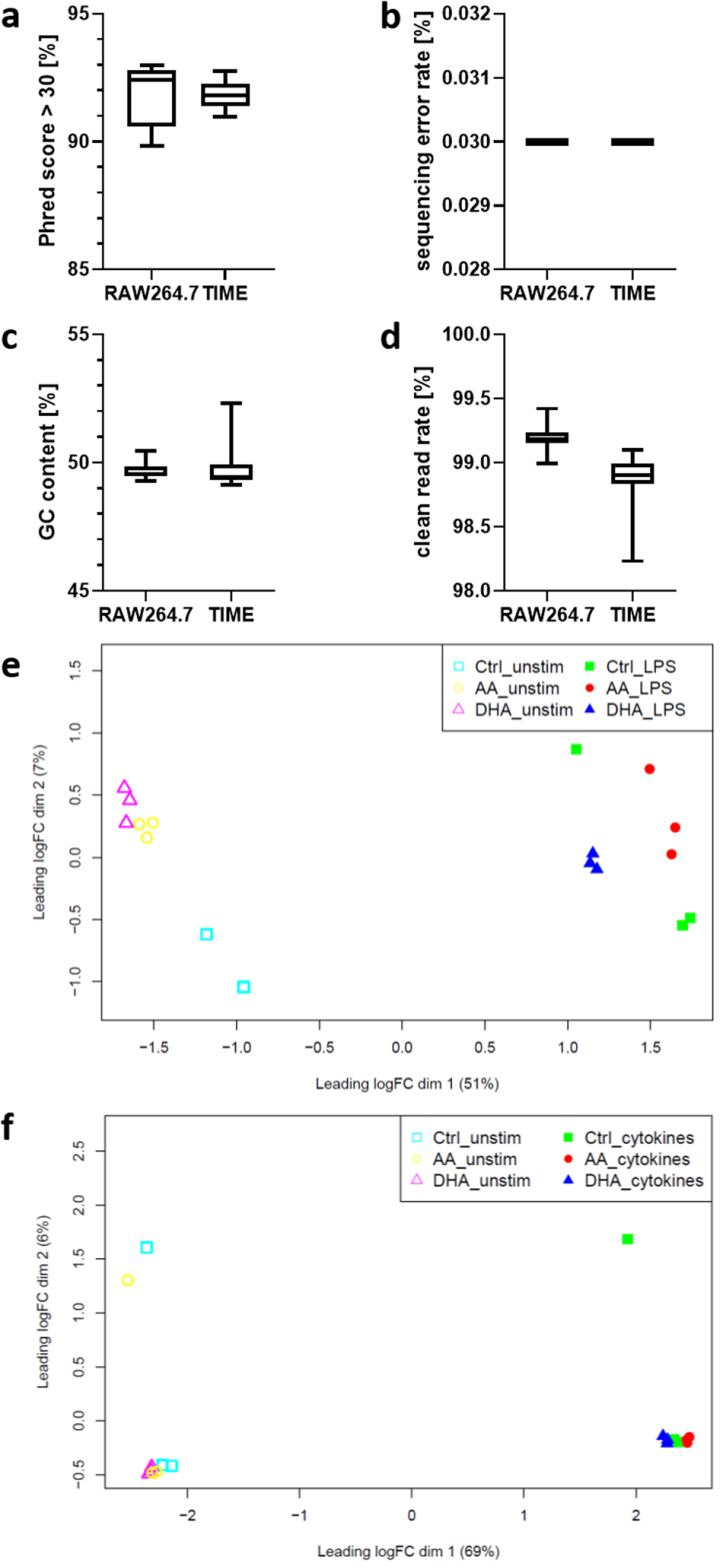
Assessment of sequence quality scores and representative MDS plots of replicate similarity. Shown for the murine macrophage cell line RAW264.7 and the human endothelial cell line TIME are (**a**) percentage of samples with a phred quality score above 30, (**b**) sequencing error rate, (**c)** GC content, (**d**) clean read rate, (**e**) multidimensional scaling within GSE142088 (RAW264.7), and (**f**) multidimensional scaling within GSE141957 (TIME).

NGS analyses were performed by experienced sequencing laboratories, namely the Core Unit DNA, University of Leipzig (GSE132361) and the Novogene (UK) Company Limited (GSE141957, GSE142088, GSE162994, and GSE162995). Quality control of the data included determination of sequencing quality (phred quality scores), sequencing error rate, GC content, and clean read rate. All quality scores were well within the acceptable range for downstream analysis (Fig. 2). Base calling accuracy proved to be of high quality with most nucleotides (>90%) in each of the reads having a Phred score greater than 30. The sequencing error rate was 0.03% throughout. GC content ranged from 49.2% to 52.3%. The ratio of clean reads was >98% for all samples. In addition, MDS plots were generated to visualize the similarity between the replicates (Fig. 2). It appeared that both the macrophage cell line RAW264.7 and the endothelial cell line TIME show a clustering of stimulated samples compared to unstimulated samples.

### RNA-seq biological replicates

Biological replicates are essential for ensuring data consistency. For this reason, all treatment conditions were replicated in at least 3 independently repeated experiments, each with a different cell passage.

## Data Availability

No custom code was used in the collection and validation of this dataset.
